# Temporal Trends in Urinary Diversion among Patients Undergoing Radical Cystectomy Between 1986 and 2022: Experience at the University Medical Center Mainz with 2224 Cases

**DOI:** 10.1245/s10434-024-15730-x

**Published:** 2024-07-05

**Authors:** Gregor Duwe, Mohamed M. Kamal, Crispin Wiesmann, Katarzyna E. Banasiewicz, Isabel Wagner, Nikita Dhruva Fischer, Maximilian Haack, Lisa Johanna Frey, Rene Mager, Thomas Höfner, Peter Sparwasser, Igor Tsaur, Christoph Wiesner, Christian Thomas, Joachim Wolfgang Thüroff, Rudolf Hohenfellner, Maximilian Peter Brandt, Axel Haferkamp

**Affiliations:** 1 Department of Urology and Paediatric Urology, University Medical Center of the Johannes Gutenberg-University Mainz, Mainz, Germany; 2 Department of Urology, Ordensklinikum Linz Elisabethinen, Linz, Austria; 3 Department of Urology, University Hospital and Faculty of Medicine Eberhard Karls University Tübingen, Tuebingen, Germany; 4 Department of Urology, Klinikum Osnabrück GmbH, Osnabrück, Germany; 5 Department of Urology, University Hospital Carl Gustav-Carus, TU Dresden, Dresden, Germany

**Keywords:** Bladder cancer, Urothelial cell cancer, Radical cystectomy, Urinary diversion, Continent urinary diversion, Incontinent urinary diversion, Time trends

## Abstract

**Background:**

Analysis of temporal trends of urinary diversion (UD) and identification of predictive factors for continent urinary diversion (CUD) in patients with bladder cancer (BC) is scarce and data on large cohorts are missing. We aimed to describe longitudinal temporal trends and predictive factors for UD among patients with BC receiving radical cystectomy (RC).

**Patients and Methods:**

We retrospectively analysed institutional data collected from patients undergoing RC from 1986 to 2022 to describe changes in patients’ characteristics and UD. Primary end points were patients’ characteristics associated with type of UD. Logistic regression analysis was used to determine predictive factors for CUD.

**Results:**

In total, 2224 patients (77.16% male, 22.84% female) with a mean age of 66 years [standard deviation (SD), 10.64 years] were included. We observed an increase in mean age from 59.86 (10.8) years (1986–1990) to 69.85 (9.99) years (2016–2022) (*p* < 0.001). The proportion of CUD gradually declined from 43.72% (94/215; 1986–1990) to 18.38% (86/468; 2016–2022). Patients who were male [odds ratio (OR): 1.92, 95% confidence interval (CI): 1.43–2.57, *p* < 0.001), younger (OR: 0.88, 95% CI: 0.87–0.89, *p* < 0.001) and had no hydronephrosis prior to RC (OR: 2.2, 95% CI: 1.66–2.92, *p* < 0.001) were more likely to receive CUD.

**Conclusions:**

We report the largest European single-center cohort of UD after RC, demonstrating a significant shift from CUD to IUD, accompanied by an increasing age. Finally, our data mirrors the development and extensive experience with the Mainz Pouch-I in the 1980’s and 1990’s together with other colon pouches.

In 2020, bladder cancer (BC) represented the tenth most common cancer worldwide with 537,278 new cases and approximately 212,536 deaths diagnosed.^1^ Overall, approximately 25% of patients with bladder cancer are diagnosed with muscle invasive bladder cancer (MIBC).^2^ Radical cystectomy (RC) is considered a treatment option for Bacillus Calmette-Guerin (BCG) unresponsive high-risk non-muscle-invasive bladder cancer and gold-standard for non-metastasized (muscle-invasive bladder cancer) MIBC.^3^ Although RC can be considered as a surgical method that is established for decades, large population-based studies in choice of UD in Europe are scarce. Meanwhile, perioperative and surgical management of BC has improved based on Enhanced Recovery after Surgery (ERAS®) pathways and robot-assisted surgery.^4,5^

Choice of urinary diversion (UD) is of major importance and decision making is commonly based on patients’ preference, clinical characteristics as well as pathological and anatomical factors (e.g. histological subtype, tumour location).^6–8^ In this context, centralization for RC in high-volume and teaching hospitals is associated with higher rates of continent urinary diversion (CUD), improved perioperative outcomes as well as postoperative morbidity and mortality.^9–11^ Interestingly, a comparison of nationwide RC databases from 2006 to 2014 between Germany and the USA revealed overall higher rates of CUD in Germany compared with the USA.^12^ This observation is consistent from large monocentric cohort studies reported from both countries.^13–15^ Moreover, these studies confirmed higher rates of CUD in high-volume and academic hospitals compared with smaller hospitals in US as well as European cohorts.^16,17^

Over the past four decades, various techniques of continent and incontinent UD have been applied at our department and, in particular, the “Mainz Pouch” (MP-) I and II were developed, resulting in one of the largest centers for RC in Germany to date.^18–20^ We sought to evaluate trends in urinary UD over a 36-year time period with historical and contemporary patient groups undergoing RC at our department. Also, we investigated changes of patients- and disease characteristics regarding to UD from 1986 to 2022.

## Patients and Methods

### Patient Population and Outcomes

We identified 2243 patients who underwent RC from January 1986 to December 2022 through a retrospectively maintained database. Overall, 19 patients were excluded owing to insufficient data. For this retrospective study, we collected patient data during regular treatment and follow-up in our department, which is permitted in accordance with the state hospital act §36, §37 of Rhineland-Palatinate. We examined how patient and disease characteristics as well as choice of UD changed over time. RC and UD were performed by open or, since being established in 2016, robot-assisted approach per patient and surgeon preference. Eligible patients underwent extended pelvic lymphadenectomy as part of institutional standard. Pathological stage was based on tumour-node-metastasis system (TNM) at RC.

We categorized UD into CUD (heterotopic and orthotopic Mainz-Pouch (MP)-I, MP-II, ileal neobladder (INB), Indiana Pouch) and incontinent urinary diversions (IUD: ileal conduit, ureterocutaneostomy, transverse colon conduit, sigmoid conduit) in order to compare the type of UD with different clinical variables. We investigated temporal changes according to patient age (including proportions of octogenarian), sex, Choice of urinary diversion, final histology and tumour stage.

### Statistical Analysis

Statistical analysis was performed using DATAtab Team (2023). DATAtab: Online Statistics Calculator. DATAtab e.U. Graz, Austria (URL https://datatab.net). Continuous variables are presented as mean ± standard deviation (SD) or medians ± interquartile range (IQR) in accordance with the data distribution. Various time trends were determined annually, or between decades. We assessed changes in UD, age and other variables over time on the basis of the data distribution, either by *t*-test, chi-squared test or analysis of variance (ANOVA). A univariate and multivariate logistic regression model was applied to identify patients’ characteristics, which predict choice of CUD. All tests were two-tailed and a *p-*value < 0.05 was considered statistically significant.

## Results

### Patient Characteristics

In total, we identified 2224 patients with sufficient clinical data available who underwent RC in our department from 1986 to 2022 (Table 1). Since June 2019, we implemented the ERAS® protocol, including various aspects in peri- and postoperative procedures, e.g. pain management, early mobilization and removal of drains.^21^ Within the total population, there were 1716 (77.16%) males and 508 females (22.84%) with a mean age of 66 years (SD: 10.64 years). Overall, 1466 (65.90%) patients underwent curative RC, while 91 (4.10%) received palliative RC. Final pathology reports revealed 1977 (88.89%) patients with urothelial cell carcinomas, while 90 (4.05%) pathology reports were not available. Eventually, the rest of the patients had rare pathological reports, such as squamous cell carcinoma, neuroendocrine carcinomas or sarcoma (Table 1). Additionally, Table 2 provides a detailed overview on temporal changes of final histology. While Ta/Tcis, T3 and T4 stages were relatively stable over time, there were stronger variations in T1 and T2 stages.

**Table 1:** Patients’ baseline characteristics, *n* = 2224

Characteristics		
Age (years)		
Mean (SD)	66 (10.64)	
Median (IQR)	67 65.56 (66.44)	

Characteristics		
	*n*	%
Sex		
Male	1716	77.16
Female	508	22.84
Urinary diversion simplified		
Continent	776	34.89
Incontinent	1406	63.22
Unknown	42	1.89
Urinary diversion		
Mainz Pouch I	610	27.40
Mainz Pouch II	80	3.60
Ileal neobladder	68	3.06
Ileal conduit	1167	52.47
Ureterocutaneostomy	86	3.87
Transverse colon conduit	66	2.97
Sigmoid conduit	46	2.07
Unknown	101	4.56
Robotic-assisted RC		
Yes	42	1.89
No	2182	98.11
Urethrectomy performed		
Yes	655	29,40
No	1344	60.48
Unknown	225	10.12
Treatment intention		
Radical RC	1466	65.90
Palliative RC	91	4.10
Unknown	667	30.00
RC final histology		
Urothelial cell carcinoma	1977	88.90
Squamous cell carcinoma	41	1.87
Adenocarcinoma	17	0.76
Sarcoma	16	0.72
Neuroendocrine carcinoma	15	0.67
No carcinoma	29	1.30
Other	39	1.73
Unknown	90	4.05
RC pathological T-stage		
T0	66	2.96
Ta/Tcis	210	9.45
T1	355	15.90
T2	491	22.10
T3	683	30.73
T4	264	11.88
Tx	6	0.27
Unknown	149	6.71
RC pathological N-stage		
N0	1447	65.06
N1	239	10.75
N2	249	11.20
N3	44	1.98
Nx	79	3.55
Unknown	166	7.46
RC pathological R-stage		
R0	1333	59.94
R1	111	4.99
R2	4	0.17
RX	14	0.63
Unknown	762	34.27
Last TURBT prior to RC		
Ta/Tcis	155	6.98
T1	630	28.73
T2	953	42.91
≥ T3	95	4.28
Unknown	391	17.10
Hydronephrosis prior to RC		
Yes	436	19.62
No	1073	48.28
Unknown	715	32.10

*IQR* interquartile range, *SD* standard deviation, *RC* radical cystectomy, *TURBT* transurethral resection of the bladder

**Table 2:** Binary logistic regression model for continent urinary diversion*, n* = 2179

Variable	Continent urinary diversion (*n* = 776)							
	Multivariate Analysis				Univariate analysis			
	OR	95% CI		*p* Value	OR	95% CI		*p* Value
		Lower	Upper			Lower	Upper	
Sex								
Female (reference)					0.4	0.33	0.49	< 0.001*
Male	1.69	1.32	2.16	< 0.001*	1.5	1.2	1.86	< 0.001*
Age								
≥ 70 years (reference)					0.13	0.11	0.16	< 0.001*
< 70 years	7.87	6.18	10.03	< 0.001*	8.21	6.52	10.33	< 0.001*
Hydronephrosis prior to RC								
Yes (reference)					0.38	0.33	0.43	< 0.001*
No	1.78	1.4	2.27	< 0.001*	2.19	1.93	2.62	< 0.001*
Time span of surgery								
2016–2022 (reference)					0.23	0.18	0.29	< 0.001*
1986–1990	1.52	1.03	2.24	0.035*	3.43	2.41	4.9	< 0.001*
1991–1995	3.26	2.21	4.8	< 0.001*	6.53	4.76	8.95	< 0.001*
1996–2000	0.94	0.62	1.41	0.761	1.92	1.37	2.69	< 0.001*
2001–2005	1.4	0.91	2.15	0.123	2.61	1.84	3.7	< 0.001*
2006–2010	2.12	1.39	3.25	**0.001**	3.08	2.17	4.35	< 0.001*
2011–2015	1.16	0.78	1.72	0.463	1.76	1.27	2.43	0.001*

*OR* odds ratio (β), *CI* confidence interval, *RC* radical cystectomy

^*^Statistically significant results

### Temporal Changes in Age and Urinary Diversion

**Fig. 1:**
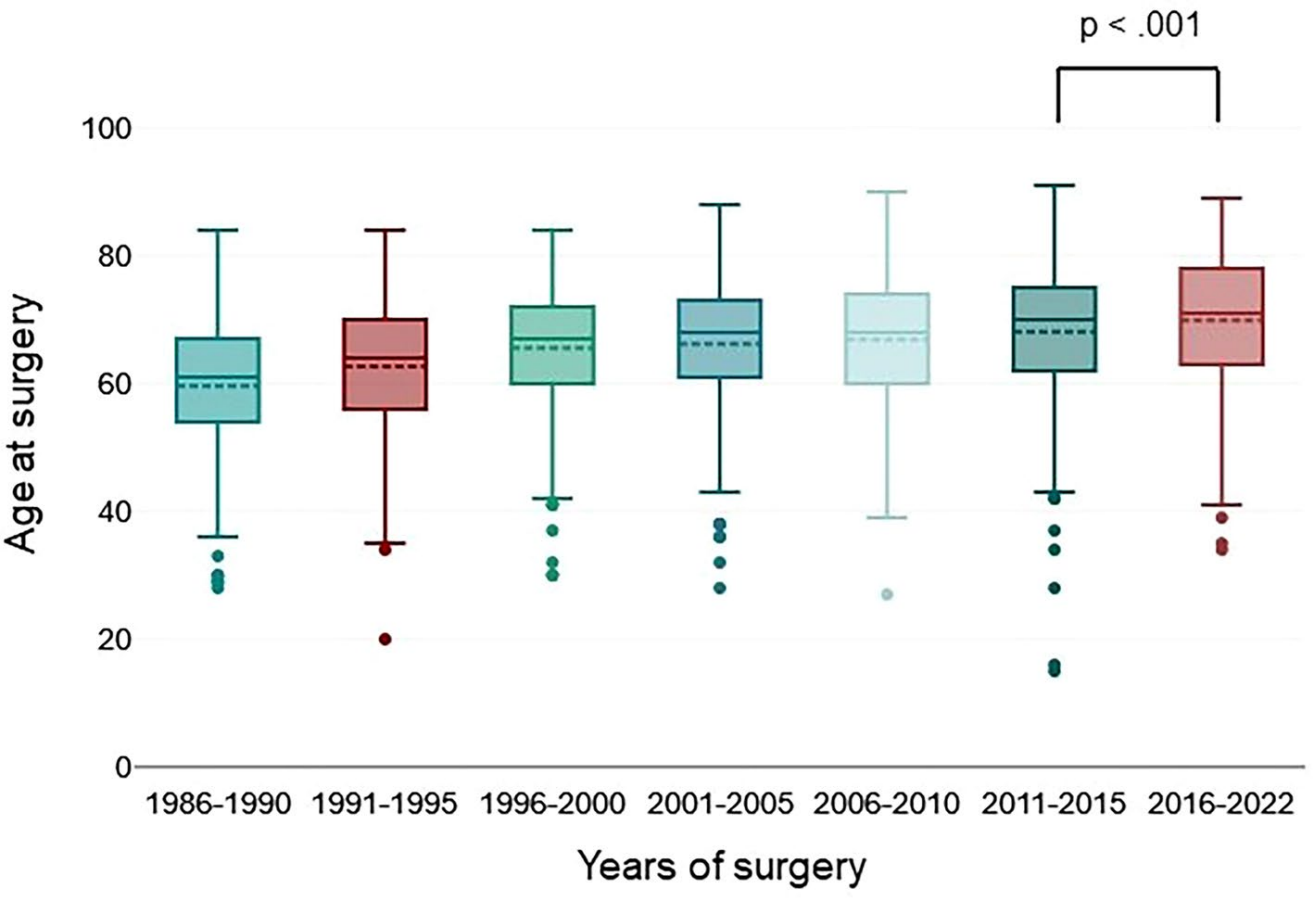
Temporal trends in mean age of patients undergoing radical cystectomy, *n* = 2224

We detected a statistically significant increase of mean age of patients receiving RC from 59.86 (SD: 10.8) years from 1986 to 1990 towards 69.85 (9.99) years from 2016 to 2022, *p* < 0.001 (Fig. 1). In particular, we described a significant increase of patients ≥ 80 years from 1.23% from 1986 to 1990 towards 17.12% from 2016 to 2022, *p* < 0.001.

**Fig. 2:**
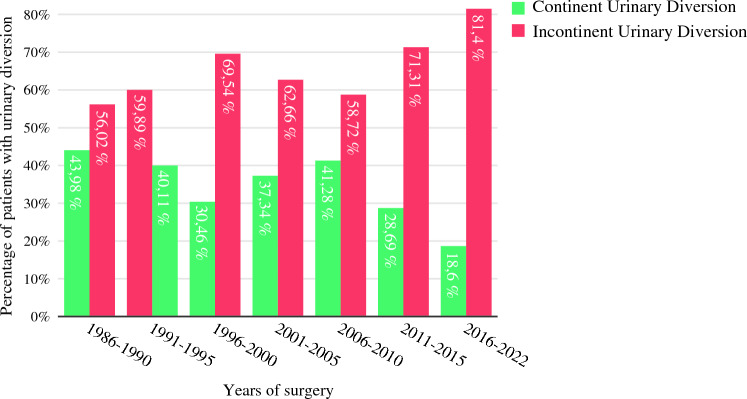
Temporal trends in urinary diversion in patients undergoing radical cystectomy, *n* = 2182, *n* = 42 were excluded owing to insufficient data on UD

**Fig. 3:**
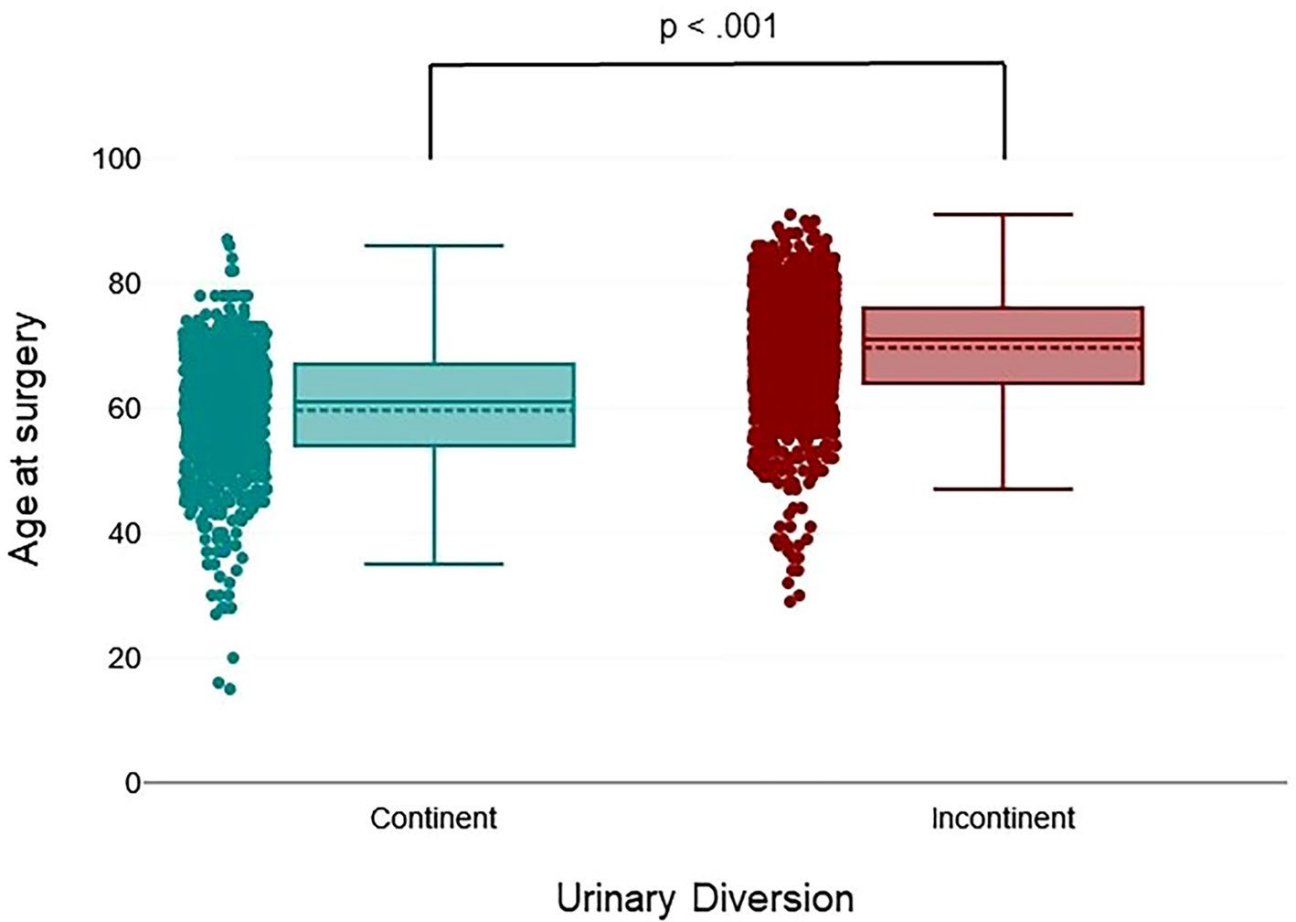
Mean age of patients with continent urinary diversions versus incontinent urinary diversions from 1986 to 2022, *n* = 2182, *n* = 42 were excluded due to insufficient data on UD

In total, we identified 776 (34.89%) patients who received continent urinary diversion (CUD) compared with 1406 (63.22%) patients with IUD (Fig. 2). Patients receiving CUD were significantly younger [59.62 (SD: 9.88) years versus 69.64 (SD: 9.26) years IUD, *p* <0.001; Fig. 3] and predominantly male (635 versus 141, *p* < 0.001). Patients ≤ 69 years received CUD (51.1%) more commonly compared with patients ≥ 70 years (11.7%; *p* <0.001).

The proportion of CUD gradually declined from 43.72% (94/215) between 1986 and 1990 to 18.38% (86/468) between 2016 and 2022 (Fig. 2). The most commonly used CUDs were the (heterotopic and orthotopic) MP-I with 610 (27.4%) patients, the MP-II with 80 (3.6%) patients and the INB with 68 (3.1%) patients. Moreover, from 1986 to 2015 the MP-I represented the only CUD performed, while the INB represented the most common CUD from 2016 to 2022 (*n* = 63/72, 87.5%). Meanwhile, the MP-II was mostly performed from 1991 to 1995 (*n* = 61/80, 76.25%). The MP-I as one of the most common CUDs, was modified using various surgical techniques.^18,20^ Ileum and caecum were primarily used for reservoir function, as well as other segments of the small intestine or colon in individual cases. Ureteral implantation was initially anti-refluxive and later primarily refluxive mechanism. Initially, the ileum invagination nipple was the primary standard continence mechanism. Over time, this was replaced by the appendix nipple. In individual cases, taped small and large bowel segments or a submucosal intestinal wall and seromuscularis tube were also used.

The IC represented the most common IUD (1167, 82.1% of all IUD; 52.5% of all RC) with a continuously increasing proportion over the past decade, followed by ureterocutaneostomy (86, 6.1%; 3.9%), transverse colon conduit (66, 3.2%; 3.0%) and sigmoid conduit (46, 3,2%; 2.1%). Patients receiving ureterocutaneostomy were significantly older with mean age of 76.23 (SD: 8.94) years followed by patients who received IC [69.92 (SD: 8.79) years, *p* < 0.001] and MP-I [59.48 (SD: 9.61) years, *p* < 0.001]. Patients who received INB were the youngest with a mean age of 61 (SD: 9.72) years, *p* < 0.001. The latest developments were characterized by the start of robot-assisted RC in 2016. While 15 robotic-assisted RC (12 IC and 3 ureterocutaneostomy) were performed between 2016 and 2020, the proportion increased towards 42 (31.6% related to 133 RC in total) robot-assisted RC (26 intracorporeal IC, 12 INB and 4 ureterocutaneostomy) between 2021 and 2022.

### Multivariate Logistic Regression Analysis on Urinary Diversion

On multivariate logistic regression analysis, receipt of CUD was associated with various patient characteristics and time of surgery. In total (*n* = 1405), male sex correlated with receipt of CUD (OR: 1.92, 95% CI: 1.43–2.57, *p* < 0.001), younger age (OR: 0.88, 95% CI: 0.87–0.89, *p* < 0.001), no hydronephrosis prior to RC (OR: 2.2, 95% CI: 1.66–2.92, *p* < 0.001) as well as time of surgery between 1991 and 1995 (OR: 2.91, 95% CI: 1.9–3.45, *p* < 0.001).

## Discussion

We present a cohort of 2224 patients undergoing RC at the University Medical Center Mainz over a 36-year period. During the 1980s, the development of the ‘Mainz-Pouch I and II’ has significantly changed the concept of UD in urology over decades in Germany, Europe and worldwide. We observed a significant increase of around 10 years of patients undergoing RC from the early 1980s to 2016–2020. The change in age distribution was accompanied by a gradual proportional decrease of CUD from 43.72% (94/215) between 1986 and 1990 to 18.38% (86/468) between 2016 and 2022. Moreover, we are presenting the largest cohort of continent ileocoecal continent urinary pouches, including 610 patients with MP- I and 80 patients with MP-II. In multivariate analysis, younger age, male sex and time of surgery during the 1980s were identified as predictive factors for receiving CUD, which is comparable to other studies.^7^

On the basis of the historical unique developments of continent ileocoecal urinary pouches, our results in trends of UD are different from other European and large US monocentric study cohorts. In a study published by Hautmann et al. in 2021 with 1100 patients undergoing RC from 1986 to 2009 in Ulm, Germany,^15^ primary CUD was INB in 816 (74.2%) cases, which can be explained by the development of the INB at this center. In our cohort, 1343 (43.41%) patients received CUD, which is significantly lower compared with Hautmann et al. One of the largest US studies is published by Bochner et al. with 2740 patients from the Memorial Sloan Kettering Center. They published data from patients receiving RC from 1995 to 2015^14^ and they reported 1797 (62%) patients receiving IC, 962 (33%) patients receiving INB and 147 (5.0%) patients receiving continent cutaneous diversion (catheterizable pouches). These 38% of CUD is comparable to our data (33.57% CUD from 1996 to 2015). The most recent and largest monocentric study was published in 2022 by Daneshmand et al. with 3347 patients treated at the University of Southern California from 1971 to 2018.^13^ In their study, 993 (29.7%) patients received IC, 1907 (57.0%) patients INB and 447 (13.3%) patients received continent cutaneous diversions. Moreover, they concordantly reported an increase in Patients age with a median age increase from 65 years from 1971 to 1979 to 71 years from 2010 to 2018. In addition, this increase in age was associated with an increase of IC from 9.1% between 1990 and 1999 to 41.7% between 2010 and 2018. Interestingly, their choice of UD significantly varied over time. While continent cutaneous UD reached a peak of 51.7% of all UD from 1980 to 1989 (4.1% from 2010 to 2018), orthotopic CUD increased from 19.0% between 1980 and 1989 towards 54.2% from 2010 to 2018. However, no detailed and precise description in temporal changes of patients age has been published in previous studies.^12–15,22^ Therefore, we consider the significant increase in patients > 80 years up to 17,12% from 2016 to 2022 relevant for the choice of UD in this patient population. Our detailed data of time trends in UD and age are not reported by Hautmann et al.^15^ and Bochner et al.^14^ owing to the different focus on survival analysis and changes in chemotherapeutic treatments. Moreover, these large numbers of CUD are also exceptionally high, reflecting their role as academic, tertiary referral centers as well as selected patients owing to their early expertise in INB.

Despite exceptionally high numbers of CUD at unifocal large tertiary referral centers, there are significant differences in choice of UD reported in population-based studies. In 2013, Kim et al. published US data from the Nationwide Inpatient Sample database of 60,187 patients undergoing RC between 2001 and 2008.^22^ They reported that only 8% received CUD with an increase from 6.6% between 2001 and 2002 to 9.4% between 2007 and 2008 (*p* < 0.001). Reported predictive factors for CUD were comparable with male sex, younger age, insurance status and treatment at teaching and high-volume hospitals. Groeben et al. compared US Nationwide Inpatient Sample data (17,711 cases) from 2006 to 2014 with German hospital billing database (60,447 cases).^12^ While the overall choice of CUD in the US remained stable at 7%, choice of CUD decreased in the German database from 36.8 to 29.2%. In multivariate analysis, age and sex were identified as the most important predictive factors for CUD. These predictive factors are again comparable to our results from multivariate analysis revealing male, younger age and time of surgery as the most important variables for receiving CUD.^7,22^

In summary, these data emphasize differences in choice of UD between the US and Germany which might be influenced by major developments in surgical techniques of CUD, in particular the Mainz-Pouch I by R. Hohenfellner (Mainz, Germany),^18^ the ileal neobladder by R. Hautmann (Ulm, Germany)^23^ and the neobladder by U. Studer (Berne, Switzerland).^24^ These circumstances have also been addressed by an international expert statement in 2015 from pioneering and leading institutions from the US and Europe by proclaiming efforts to achieve higher rates of CUD on the basis of better definition ‘of the quality-of-life impact, technique dissemination and the centralization of RC which should not be the exclusive domain of large cystectomy centers’.^25^ In future, we expect new challenges in optimal patient selection for best choice of UD^26^ owing to the increasing rates of robot-assisted RC.^27^ The development of robotic-assisted RC was first accompanied by higher rates of IUD which is demonstrated in a US national wide analysis from 2004 to 2013 among 27,170 patients reported CUD rates of only 10.2% in those centers performing ≥ 75% minimally invasive RC.^28^ Furthermore, the increasing establishment of ERAS® protocol for RC, which we have implemented in our department in 2020,^21^ might have contributed to safer surgery of our elderly and octogenarian patients, potentially leading to higher rates of IUD. Undoubtfully, there is a significant impact of high-volume hospitals, even superior to surgeon volume,^17,29^ on improved surgical and oncological outcome accompanied by CUD that is even superior to single surgeon volume.

Our study has limitations that warrant consideration. Owing to the study’s retrospective nature, we are not able to provide broad data of patients’ characteristics and their follow-up data. Despite our efforts to receive all available survival data of our patients from the cancer registries, we have no sufficient long-term survival data. Therefore, we were only able to conduct analysis of clinical variables which were defined by available data from maintained patients’ charts. Thus, data of comorbidities, performance status and oncological treatments, including chemotherapy and targeted therapies, are not available for the overall cohort. These circumstances might lead to a population and selection bias owing to the study’s retrospective nature, e.g., exclusion of 42 patients in analysis of UD time trends owing to the lack a data. Notably, availability of clinical data from patients treated in the 1980s up to 2010s was almost impossible to retrieve sufficiently. Clinical factors, such as serum creatinine, pelvic muscle status, Charlson comorbidity index, body mass index or prior abdominopelvic radiation or surgery, might compromise our results and conclusions. Nevertheless, the strength of the study relies on the largest European single-center cohort of RC. Our data reflect the historical and surgical developments in RC from 1986 onwards which has contributed significantly to various techniques in CUD, in particular the Mainz-Pouch I and Mainz-Pouch II by Rudolf Hohenfellner as the founder and first director of our department.^18,19^ Throughout the entire time of our study cohort, standards for clinical decision making, indications for RC and choice of UD as well as technical and surgical principles have remained consistent by a group of high-volume surgeons. Thus, our population analysis on a decade-by-decade basis allows the reflection of evolution of surgical management in RC and UD in the context of historical and contemporary developments.

## Conclusions

To our knowledge, we are presenting the largest European single-center cohort of urinary diversion after RC with consistency of standardized surgical techniques and philosophy. Over the 36-year period, our data reflects a significant temporal shift away from CUD to IUD, accompanied by an increase in patients’ age which might be explained by a demographic change. Finally, our data represent the development and extensive experience with the MP-I and other colon pouches. In future, we expect a further increase of robotic-assisted RC.
